# The risk of bias in observational studies of exposures (ROBINS-E) tool: concerns arising from application to observational studies of exposures

**DOI:** 10.1186/s13643-018-0915-2

**Published:** 2018-12-21

**Authors:** Lisa Bero, Nicholas Chartres, Joanna Diong, Alice Fabbri, Davina Ghersi, Juleen Lam, Agnes Lau, Sally McDonald, Barbara Mintzes, Patrice Sutton, Jessica Louise Turton, Tracey J. Woodruff

**Affiliations:** 1 Charles Perkins Centre and School of Pharmacy, Faculty of Medicine and Health, The University of Sydney, D17, The Hub, 6th floor, Sydney, New South Wales 2006 Australia; 2 School of Medical Sciences, Faculty of Medicine and Health, The University of Sydney, Sydney, Australia; 3 National Health and Medical Research Council, Canberra, Australia; 4 Department of Ob/Gyn & the Institute for Health Policy Studies, University of California, San Francisco, USA; 5 Department of Health Sciences, California State University, East Bay, San Francisco, USA; 6 School of Pharmacy, University of California, San Francisco, USA; 7 Charles Perkins Centre, The University of Sydney, Sydney, Australia; 8 School of Pharmacy, Faculty of Medicine and Health and Charles Perkins Centre, The University of Sydney, Sydney, Australia

**Keywords:** Systematic review, Risk of bias, Quality assessment, Public health guidelines, Guidelines, GRADE, Cochrane, Nutrition, Environment, Observational study

## Abstract

**Background:**

Systematic reviews, which assess the risk of bias in included studies, are increasingly used to develop environmental hazard assessments and public health guidelines. These research areas typically rely on evidence from human observational studies of exposures, yet there are currently no universally accepted standards for assessing risk of bias in such studies. The risk of bias in non-randomised studies of exposures (ROBINS-E) tool has been developed by building upon tools for risk of bias assessment of randomised trials, diagnostic test accuracy studies and observational studies of interventions. This paper reports our experience with the application of the ROBINS-E tool.

**Methods:**

We applied ROBINS-E to 74 exposure studies (60 cohort studies, 14 case-control studies) in 3 areas: environmental risk, dietary exposure and drug harm. All investigators provided written feedback, and we documented verbal discussion of the tool. We inductively and iteratively classified the feedback into 7 themes based on commonalities and differences until all the feedback was accounted for in the themes. We present a description of each theme.

**Results:**

We identified practical concerns with the premise that ROBINS-E is a structured comparison of the observational study being rated to the ‘ideal’ randomised controlled trial. ROBINS-E assesses 7 domains of bias, but relevant questions related to some critical sources of bias, such as exposure and funding source, are not assessed. ROBINS-E fails to discriminate between studies with a single risk of bias or multiple risks of bias. ROBINS-E is severely limited at determining whether confounders will bias study outcomes. The construct of co-exposures was difficult to distinguish from confounders. Applying ROBINS-E was time-consuming and confusing.

**Conclusions:**

Our experience suggests that the ROBINS-E tool does not meet the need for an international standard for evaluating human observational studies for questions of harm relevant to public and environmental health. We propose that a simpler tool, based on empirical evidence of bias, would provide accurate measures of risk of bias and is more likely to meet the needs of the environmental and public health community.

## Background

Public health guidelines (e.g. drinking water quality, dietary, environmental hazard and risk assessments) have a direct, long-term impact on health. Systematic reviews are increasingly required for these types of guidelines [1–4]. Systematic review methods are also becoming more prevalent in research areas that rely on observational studies of exposures to assess harm [1, 2, 5–9]. In nutrition research, for example, it is not feasible to investigate the effect of a particular food or nutrient on chronic disease incidence using a controlled study design because these conditions (e.g. cardiovascular disease, bowel cancer) take several decades to develop and/or become symptomatic. In environmental health, human observational data are usually the most directly applicable data available because ethical considerations virtually preclude human randomised controlled trials (RCTs).

A critical step in the systematic review process is the assessment of the risk of bias of included studies. Risk of bias, which is analogous to internal validity, assesses whether flaws in the design, conduct or analysis of a study may lead to biases that affect the results [10]. Since environmental and public health guidance is primarily based on evidence from human observational studies, a risk of bias tool that can be applied to such studies is needed. Although many tools exist, they have often been developed for one or a few specific systematic reviews, are inadequately described, and lack evaluation [11, 12]. There is currently no universally accepted standard or consensus about the best approach for assessing risk of bias in observational study designs. This can make both systematic reviews and public health guidelines difficult to interpret and evaluate because they use different methods.

Most of the efforts to reduce bias in guideline development have focused on clinical practice guidelines, and some guideline developers adopt methods used to evaluate clinical research to assess observational studies [13]. The Cochrane tool for assessing risks of bias in randomised controlled trials is widely used for clinical systematic reviews and guideline development [14, 15]. An international group of epidemiologists, statisticians, systematic reviewers, trialists and health service researchers developed the ROBINS-I (‘Risk of Bias in Non-randomized Studies of Interventions’) tool building upon developments in risk of bias assessment of randomised trials and diagnostic test accuracy studies [14, 16]. ROBINS-I is based on the premise that an observational study of an intervention should be compared to a hypothetical randomised controlled trial to identify potential biases [17].

Environmental and many other public health studies, such as dietary or health behaviour studies, do not test interventions. Rather, they observe whether there is an association between an exposure not under the investigator’s control and a health outcome. In these cases, it may be considered most appropriate to assess risk of bias using an appraisal tool that is specific to studies of exposures, not interventions. As part of a programme of work to adapt Grades of Recommendation, Assessment, Development and Evaluation (GRADE) for environmental health, an international group of researchers modified the ROBINS-I tool to develop ‘The Risk of Bias in Non-randomized studies of Exposures’ tool, called ROBINS-E [version July 2017] [7, 18]. Like ROBINS-I, ROBINS-E compares the study being evaluated to a hypothetical, ‘ideal’ randomised, controlled trial [19]. ROBINS-I was modified to ROBINS-E by replacing the term ‘intervention’ with ‘exposure’; renaming of ‘target trial’ to ‘target experiment’; adding fields to collect information on measurement of exposures and outcomes and adding questions to assess bias in exposure measurement [19]. The ROBINS-E tool assesses 7 domains of bias: confounding, selection of participants into the study, classification of exposures, departures from intended exposures, missing data, measurement of outcomes and selection of the reported result. Within each of these domains, ‘signalling questions’ are asked to aid the user in making judgements. Lastly, judgements within each domain are summarised into an overall risk of bias assessment for each study.

The ROBINS-E tool remains under development, and further refinements are not expected to change the domains of bias assessed [19]. Therefore, this is an appropriate time to gather experience with the practical application of the tool to exposure studies. Although ROBINS-I has been evaluated for intervention studies [20, 21], to our knowledge, this is the first paper summarising user experience with ROBINS-E. As a result of our concerns, we encourage the development of a tool that incorporates existing empirical evidence on the aspects of observational study design that potentially bias outcomes.

## Methods

This paper reports our experience of the practical application of ROBINS-E [18] to 74 exposure studies in 3 areas: dietary exposure, drug harm and environmental exposure. Twelve researchers (the authors) were convened to reflect the diverse range of backgrounds that might be found among potential users of the tool. Highest degrees included PhD, MD, PharmD and Master’s degrees in disciplines including public health, epidemiology, environmental health, nutrition and clinical medicine. Relevant work experience ranged from 1 to 27 years. The team included researchers whose first language is not English. All authors have conducted risk of bias assessments in the context of systematic reviews and 4 authors (LB, DG, BM and TW) also have experience in conducting risk of bias assessments in the context of developing guidelines, risk assessments, or other normative guidance that include observational studies of exposures.

We conducted a pilot test of ROBINS-E [version July 2017] [18] to discuss and clarify varying interpretations of the questions. Seven of the authors (LB, NC, JD, AF, DG, AL, BM) applied ROBINS-E to 3 observational studies from different research areas relevant to meta-analyses that we were conducting (1) a cohort study examining association of dairy consumption with cardiovascular disease [22], (2) a nested case-control study examining association of drug exposure (domperidone) with ventricular arrhythmia [23] and (3) a cross-sectional study examining the association of wind turbine noise with sleep and health outcomes [24]. Based on our experience with this pilot, we clarified questions and developed supplemental guidance for our teams of coders. For example, our supplemental guidance provided definitions for ‘valid and reliable’ exposure and outcome measurements and indicated questions that could be ignored because they did not apply to exposure studies (e.g. regarding ‘intention to treat’). To promote consistency in how the questions were answered, we also created decision rules for some questions. For example, we agreed that question 7.1 regarding bias in the selection of results would be rated as low risk of bias only if a study protocol could be obtained and it could be determined that all collected results were reported.

We applied ROBINS-E [18] to studies included in systematic reviews we are conducting on nutrition, drug harm and environmental topics. Seventy-four studies were double-coded by teams of 2 authors (NC, AF, AL, BM, SM, JT). Following usual procedures for risk of bias assessment [10, 14, 17], the coders reached consensus on their judgements for each domain. If consensus could not be reached, a third author adjudicated. The assessed studies examined the association of dairy consumption with cardiovascular outcomes (*n* = 42; 37 cohort studies, 5 case-control studies), grain consumption with cardiovascular outcomes (*n* = 24; 21 cohort studies, 3 case-control studies) and cardiac risks of domperidone exposure (*n* = 8 studies with 9 analyses; 2 cohort studies, 4 case-control studies, 3 case-crossover studies, with one study reporting both a case-control and case-crossover analysis, each of which was assessed separately for risk of bias). Data from the risk of bias assessments were entered into a data extraction form using REDCap electronic data capture tools hosted at The University of Sydney [25]. The risk of bias assessment for each paper will be reported in the systematic review in which it is included.

This paper reports user feedback on the ROBINS-E. After the completion of the pilot study and coding of all articles, each coder provides written feedback structured according to each question in the tool, with an additional section to collect any overall feedback. Coders were encouraged to provide specific examples from studies to supplement their feedback. In addition, the process of applying ROBINS-E was discussed in a combined face-to-face video conference meeting among the coders, and the discussion was documented in writing by LB. The discussion was structured by reviewing each question in the tool and documenting comments by questions, and then documenting overall comments on the tool. We discussed the domain elements, accuracy and clarity of each question, and the overall ease of use, including the time it took to complete the assessments. The individual comments and documented discussion were distributed to all coders as Word documents. We inductively and iteratively classified the feedback from the individual coders and group meeting into 7 themes based on commonalities and differences until all the feedback was accounted for in the themes. All authors were then given the opportunity to review the themes and suggest edits. The final themes are listed in Table 1 and summarised below.

**Table 1:** ROBINS-E user experience themes and concerns

1. Comparison to an ‘ideal’ randomized controlled trial (RCT)	
RCTs are not available for exposure studies and, therefore, not relevant to decision makers who must rely on observational studies of exposures.	
Assessing observational studies based on RCTs results in a default rating of high risk of bias	
Some of the questions derived from evaluating RCTs of interventions are inappropriate or impossible to apply for observational studies.	
Sources of bias specific to observational studies may not be captured by comparison to an RCT.	
2. Inadequate assessment of bias related to confounding	
Does not capture bias related to over-adjustment for confounders or inappropriate modelling of confounders.	
Does not capture advantages of newer statistical methods used for control for confounding.	
Clearer guidance is needed on method for identifying confounders.	
Does not differentiate between confounders, co-exposures and complex exposures.	
3. Inadequate assessment of bias related to measurement of exposure	
Assessment is limited to validity and reliability of the measurement, and these concepts are not clearly defined.	
4. Use of an overall risk of bias rating	
Does not distinguish between studies that have a ‘serious’ risk of bias in one domain and those that have multiple ‘serious’ risks of bias.	
Assumes all risk of bias domains are weighted equally.	
5. Additional risks of bias relevant to observational studies are not assessed (e.g. funding source)	
6. Signalling questions	
Do not consistently help the raters come to a consensus on how to rate a bias domain.	
Specific questions unclear or confusing.	
7. Practical considerations	
Time-consuming to use.	
There are limitations of using a single tool to rate different study designs.	

## Results

Table 1 lists the 7 themes derived from the user feedback and the major concerns related to each theme. Each theme is discussed in more detail below.

### Comparison to an ‘ideal’ RCT

ROBINS-E, like ROBINS-I, is based on a structured comparison of the observational study being rated to a hypothetical ‘ideal’ randomised controlled trial [26]. The process of using ROBINS-E begins with creating the ideal randomised trial specifying the population to be studied, the exposure assessed, the comparison to the exposure and the outcomes to be measured.

There are some advantages to this approach. ROBINS-E identifies key features of RCTs that reduce bias compared to observational studies and asks questions related to these key features. For example, randomisation theoretically eliminates confounding and ROBINS-E asks a series of questions to determine how likely it is that uncontrolled confounding has influenced the observational study result. Blinding in an observational exposure study minimises observer and reporter bias in the measurement of exposure and outcome. ROBINS-E usefully assesses these biases by asking for cohort-type studies: ‘Were outcome assessors unaware of the exposure received by study participants?’ and for case-control studies: ‘Was the definition of case status (and control status, if applicable) applied without knowledge of the exposure received?’

The ideal RCT is used as the comparison because it is at the top of an evidence hierarchy organised by increasing protection against bias. But, the relative value of observational and experimental studies also depends on the question [27]. Observational studies are the best design for answering questions aimed at assessing harm from exposures because real-world exposures are often complex and are never controlled by the investigator. Observational studies do not consistently find different effect estimates than RCTs, suggesting that multiple sources of bias can influence effect estimates of observational studies or RCTs [28, 29]. The ROBINS guidance indicates that the target trial ‘need not be feasible or ethical’ [17]. In the case of studies designed to evaluate potentially harmful exposures, the target trial could never be designed for a combination of ethical and practical reasons. For example, if a chemical is suspected of being carcinogenic, it would be unethical to randomise trial participants to exposure, and both the number of participants and duration of exposure required would make such a trial impractical. Thus, RCTs will not be available for systematic reviewers and decision makers who need to address questions of harm.

Additional limitations exist because some of the questions derived from evaluating RCTs are inappropriate or impossible to apply for observational studies. For example, ROBINS-E considers biases that arise due to departures from intended exposures as performance biases. They arise when differences occur after the start of interventions in RCTs or exposures in observational studies, but the participant continues (for analysis purposes) to be part of the intended intervention or exposure group. In randomised trials, performance bias can be minimised by blinding of participants and providers of the intervention. ROBINS-E addresses performance bias by asking questions about co-exposures, contamination, switches and fidelity of implementation. As the exposures being measured are unintended and are not controlled by the investigator, concepts such as switching and fidelity of implementation do not generally apply to observational studies of exposure. For example, the question regarding ‘deviation from intended exposures’ cannot be answered, as exposures are never intended. This question only makes sense in the context of an RCT of an intervention. For case-control studies, this ‘ideal’ RCT framework is particularly unhelpful as a tool to inform risk of bias evaluations due to their retrospective study design, and the use of this design to assess infrequent serious health outcomes.

In addition, there are potential sources of bias that might afflict a particular type of observational study that are not identifiable by comparing it with a theoretical RCT. For example, failure to match by risk set in a nested case-control study or control for confounding with the matching variable in a matched case-control study can induce bias. In addition, in an RCT, the start of exposure is clearly defined. In exposure studies, the more crucial question is whether follow-up begins at initiation of exposure and this is not assessed by ROBINS-E. These real and important sources of bias specific to aspects of observational study design cannot be detected and assessed by comparing these studies to the theoretic RCT framework of the ROBINS-E tool.

Lastly, the RCT framework does not consider advantages that an observational design can have over a randomised design. Exposure studies often include a broad gradient of exposure levels, unlike trials that are often limited to only a few comparison groups. This range of exposure levels allows dose-response relationships to be established. Dose-response relationships are an important consideration for determining true associations between exposures and health outcomes because of the improbability that bias, except due to confounding with a closely related variable, would mirror the dose-response relationship. Furthermore, ROBINS-E does not assess bias in how dose-response relationships are established because exposure levels are only considered as one aspect of whether measurements of exposure were ‘robust’ [18].

### Inadequate assessment of bias related to confounding

#### Determining if uncontrolled confounding biases outcomes

Assessing bias related to confounding is important for observational studies. Confounders are defined as factors that are associated with the exposure and prognostic for the outcome, but are not on the causal pathway [30]. ROBINS-E has limitations in determining whether confounders will bias study outcomes. ROBINS-E rates a study as having a high risk of bias if it does not control for any or enough relevant confounders. However, there is no question in ROBINS-E regarding the potential to introduce bias through controlling for large numbers of baseline confounders unnecessarily (over-adjusting) [31].

Assessment of the risk of bias associated with confounding reflects not only whether a specific confounder such as age is included in a study, but also how that confounder is modelled. The use of very broad age categories could lead to a serious risk of bias, for example, in a study that assessed cardiac risks of a specific exposure and compared groups with unequal age distributions. Additionally, many newer studies use tools such as propensity scores (or high dimensional propensity scores) to account for confounding. ROBINS-E provides inadequate guidance to assess how confounders are modelled or the application of these tools.

#### Identifying confounders

One of the strengths of the ROBINS-E is that prior to beginning the risk of bias assessment, the investigator is required to pre-specify relevant confounders. This means that all studies will be evaluated for methods used to control or account for the same set of confounders.

The ROBINS-E provides some guidance for identifying confounders, stating that critical confounders ‘are likely to be identified both through the knowledge of the subject matter experts who are members of the [systematic] review group, and through initial (scoping) reviews of the literature’. The guidance should also recommend that other experts who are not part of the review group—such as epidemiologists, toxicologists, biostatisticians, systematic review experts and biologists—should be consulted. This wider consultation with experts in the field should be conducted in a systematic and comprehensive way (e.g. [32]).

Ideally, confounders should be identified by searching for systematic reviews examining the association of potential confounders with relevant outcomes and assessing the quality of the reviews using a tool such as ROBIS (Risk of Bias in Systematic Reviews) [33]. This is a more rigorous method than the one recommended by ROBINS-E. However, applying this method consistently for all outcomes and bodies of observational studies would require substantial time. For our application of ROBINS-E, we consulted experts in the field relevant to each review we were conducting and identified systematic reviews that verified whether a particular variable was a confounder. See Table 2 for identification of confounders for each outcome assessed in studies evaluating the association of dairy consumption with cardiovascular disease. Although this list is based on published systematic reviews, we did not assess the risk of bias of each review identified as this would have been too time-consuming. Instead, we relied on the most recent published systematic reviews that appeared to have conducted a comprehensive search. Even so, it took over 2 weeks to create and agree upon the list of confounders for the review evaluating the association of dairy consumption with cardiovascular disease. Greater resources would be needed to identify beforehand the confounders for broader questions, such as ‘What are the adverse health effects of living near a waste dump’ or ‘living near a wind farm’. These questions are relevant for public health guidelines, but may consider a very broad range of outcomes including developmental, psychological or clinical outcomes, and their relevant confounders. The practical limitation of using a rigorous method to identify potential confounders must be balanced against using a less rigorous method, such as expert opinion, which makes the selection of confounders more subjective.

**Table 2:** Table of critical confounders developed for a systematic review of studies assessing the association of dairy intake with cardiovascular outcomes

Outcome	Confounders (p/h)	Confounders (all outcomes)
1. CVD mortality	Fibre supplement (p) Red meat (h) Sodium (Na+) (h)	Age Sex BMI Smoking Alcohol intake History of co-morbidities Parenteral/Fhx MI < 60 years PA levels SES Total energy intake Fruit and vegetable intake *Specialised confounders* Hormone therapy
2. CVD events	Fibre supplement (p) Magnesium supplement (p)	
3. CHD mortality	Fibre supplement (p) Trans fat (h) Polyunsaturated fat (n-6) (p) Sodium (+Na) (h)	
4. CHD events	Fibre supplement (p) Trans fat (h) Magnesium supplement (p) Polyunsaturated fat (n-6) (p)	
5. Total MI	Aspirin (p) Vitamin E supplement (p)	
6. Fatal MI	Vitamin E supplement (p)	
7. Non-fatal MI	Aspirin (p)	
8. Total stroke	Potassium supplement (p) Red meat (h) Sodium (+Na) (h)	
9. Ischemic stroke	Aspirin (p) Polyunsaturated fat (LC n-3) (p) Red meat (h)	
10. Haemorrhagic stroke	Aspirin (h)	

*p* protective, *h* harmful

#### Co-exposure vs confounding confusion

ROBINS-E defines co-exposures as ‘exposures that individuals might receive after or with initiation of the exposure of interest, which are related to the exposure received and which are prognostic for the outcome of interest.’ During our application of ROBIN-E, the term co-exposure caused confusion. In public and environmental health, most exposures are complex, so the exposure of interest is composed of multiple co-exposures. For example, fumes in a nail salon contain toluene, formaldehyde, phthalates and methyl acrylates, among other chemicals. The distinction between co-exposures and confounders is less relevant in observational studies as co-exposures are usually considered as confounders and, when appropriately adjusted, can better represent real-world complex exposures. Misclassification of exposure is more of a concern in observational studies than the contamination of the different exposure groups [31]. Because ROBINS-E was derived from a tool to assess intervention studies, it does not clearly differentiate between confounders, co-exposures and complex exposures.

Most importantly, in the context of observational studies, co-exposures may be the same as confounders. ROBINS-E distinguishes between characteristics and exposures that are present at baseline, which are defined as confounders, and additional exposures that occur at the same time or following initiation of the exposure of interest. These additional exposures are defined as co-exposures. In practice, this distinction is often arbitrary, as many exposures can be present at baseline and/or after initiation of the exposure of interest. In studies examining the association of whole grain breakfast cereal with cardiovascular outcomes, milk consumption at baseline is a confounder because it is associated with the exposure and prognostic for the outcomes, but not on the causal pathway. But, as breakfast cereal may be eaten with milk, it could also be considered a co-exposure under the ROBINS-E definition because it is received with the exposure of interest and prognostic for the outcome of interest. For a study of cardiac harms of domperidone, exposure to another medication that prolongs the QT interval could be considered a confounder as it may be associated with exposure (e.g. domperidone-treated patients may also be more likely to receive this drug) as well as cardiac risks. However, it could also be considered a co-exposure, with additional analyses carried out to explore whether there are interaction effects. These interaction effects would not be expected to differ depending on whether the QT-prolonging medication had first been prescribed before domperidone, at the same time, or afterwards, as long as a person was exposed to the two drugs concurrently. In the case of complex exposures (such as the various nutritional components of dietary dairy exposure, or chemical mixtures), co-exposures should not be modelled separately, but would instead be a component of the description of the exposure under assessment.

Controlling for co-exposures that are not confounders, as suggested by ROBINS-E, could induce bias. When analysing presumed causes and effect, including variables that are not known to be confounders (i.e. correlated with both exposure and outcome) and controlling them as confounders could result in over-adjustment of the model, a loss of power and a bias towards the null. Likewise, inappropriately adjusting a variable that lies in the causal pathway between the exposure of interest and outcome as a confounder will bias the effect of the exposure of interest towards the null. Exclusion of cases with co-exposures can also lead to a biased effect estimate if the co-exposure is not associated with the outcome. In sum, there is no analytic reason to evaluate co-exposures if they are not associated with the outcome or can be considered as confounders.

### Inadequate assessment of bias related to measurement of exposure

Determining error in the measurement of exposures and confounders is critical to assessing risk of bias in a study. The ROBINS-E tool asks investigators to specify the methods used to measure these variables and to determine if exposures and outcomes are ‘measured validly and reliably’. We found it necessary to pre-specify the criteria we would use to rate a method as valid. For example, dietary questionnaires are frequently used to assess dietary intake as an exposure. We specified that Food Frequency Questionnaires would be considered low risk of bias for validity of measurement if the study reported that the tool was validated in another study, with the reference provided and relevant coefficients reported.

By limiting the assessment of an exposure measurement to its validity and reliability, ROBINS-E may not adequately capture other deficiencies in measurement that could contribute to bias. ROBINS-E does not assess details of exposure measurement which could be related to the outcome, including the dose, duration or developmental stage at which the exposure occurs. ROBINS-E does not consider differential biases in exposure measurement across study participant groups. Such biases in measurement of air pollution exposures, for example, could result in attenuation of the observed results. Furthermore, surrogate measures such as distance to freeway can often create systematic biases. There have been some efforts to develop instructions tailored to exposures relevant to the study question beforehand, such as for case studies involving air pollution exposures [34].

### Inappropriate use of an overall risk of bias rating

The ROBINS-E guidance states that the overall rating for risk of bias is determined by the highest risk of bias rating for an individual domain. This rating system implies that all domains contribute equally to the risk of bias of the overall study. It also means that a study with a ‘serious’ risk of bias in one domain is rated similarly to another in which nearly all domains are judged to be at serious risk of bias, thus failing to discriminate between studies with different biases. Similarly, ‘quality scores’ have not been able to distinguish between high and low risk of bias studies in meta-analyses [35] and there is no empirical evidence to support how each risk of bias item should be weighted [10, 36]. Therefore, the ratings of each domain of the tool are typically reported for each study, allowing users to clearly identify the different sources of bias in a study.

### What is missing in the ROBINS-E risk of bias assessment?

The ROBINS-E tool is based on a narrow definition of bias: an error in quantitative effect estimates that may result from a methodological flaw. Non-methodological characteristics can also influence effect estimates and the inferences drawn from them. Two potential sources of bias that are important for exposure studies were not assessed with the ROBINS-E tool: funding sources and conflicts of interest of investigators. Evidence across a variety of fields shows that industry sponsorship is associated with outcomes that favour the sponsor’s product, even when industry- and non-industry-sponsored studies have similar methodological risks of bias [37, 38]. In studies of harmful exposures, industry sponsorship is generally expected to be associated with a bias towards the null.

### ‘Signalling questions’ not linked to risk of bias ratings in each domain

Each domain lists signalling questions to facilitate judgements about the risk of bias in each domain. Our raters agreed on the domain ratings most of the time, but often disagreed on how they rated the signalling questions. Although the signalling questions are useful for making the rationale behind the assessment of each domain transparent, they do not help the raters come to a consensus about what they should be considering under each domain. Our raters noted that even when they differed on their answers to the signalling questions, they could have the same rating for the bias domain. Thus, the reasoning behind their ratings was not adequately captured by the signalling questions. The manual does not indicate whether the answers to the signalling questions need to be resolved. Additionally, inadequate guidance is provided on the link between responses to multiple signalling questions within a domain and the risk of bias assessment for the domain. It was not clear to the raters whether a single signalling question indicating a high risk of bias should result in the risk of bias for the domain being rated as ‘serious’. Specific issues related to answering the signalling questions for exposure studies are described in Table 3.

**Table 3:** Comments on signalling questions in the ROBINS-E risk of bias tool that were difficult to assess and often irrelevant to a particular study

*Domain 1: Confounding*	
*Signalling question 1.3: time-varying confounding:* Were exposure discontinuations or switches likely to be related to factors that are prognostic for the outcome?	
Cohort studies can continue over decades so changes in exposure may be related to a wide variety of factors. For example, in studies assessing dietary exposures, it is impossible to distinguish whether someone has made a change in their diet due to a diagnosis or onset of a symptom rather than personal choice or social reasoning (e.g. veganism).	
*1.4: Baseline confounding:* Did the authors use an appropriate analysis method that adjusted for all the critically important confounding areas?	
Most of the studies we coded had many relevant confounders, and it was rare that all confounders were controlled in every study, so we modified this question by developing decision rules around the number of confounders that were taken into account. We also determined if the study avoided adjusting for post-exposure variables. For example, in a study assessing cardiovascular disease (CVD) as an outcome, it is inappropriate to adjust for new incidence of hypertension that has occurred during the exposure period. Hypertension is not a confounder because it is on the causal pathway to CVD.	
*Domain 2: Bias in selection of participants*	
*2.3 and 2.3:* Were the post-exposure variables that influenced selection associated with exposure? Were the post-exposure variables that influenced eligibility selection influenced by the outcome or a cause of the outcome?	
Since cohort studies are often assembled based on exposure levels, it is rare for selection to be unrelated to exposure. In exposure studies, participants are almost always selected into the study based on characteristics that are assessed after the start of exposure. For example, in a study assessing the association of an exposure with cardiovascular disease, subjects may be excluded if baseline surveys or clinical records determine they have diabetes, hypertension or metabolic syndrome, characteristics which may be associated with exposure or outcome.	
*Domains 3 and 4: Exposures*	
*2.4* Do start of follow-up and start of exposure coincide for most participants? *3.2* Did entry into the cohort begin with start of the exposure?	
For many types of exposures, such as dietary exposures or various types of pollution, exposure can begin in infancy, long before entry into a cohort. Unlike interventions, exposures are not initiated by the investigators, so exposure and follow-up will rarely coincide.	
*4.1* Is there concern that changes in exposure status occurred among participants? *4.2* Did many participants switch to other exposures?	
In exposure studies, there is always a concern that changes in exposure status occurred among participants. It is rare that exposure measurements are made continuously over long periods of exposure. Techniques are used that are likely to correct for this issue, such as multiple assessments of exposure (e.g. every 2 years) and person-years adjustment. ROBINS-E terms such as ‘intended’ exposure, ‘initiating and adhering to an exposure’ and ‘switching’ exposures are applicable to randomised trials, but do not apply to exposure studies where exposure is not controlled by the investigators.	
*Domain 5–7:* Bias due to missing data, bias in measurement of outcomes and bias in the selection of reported results	
Most of the questions related to these domains were applicable to observational studies. Signalling questions related to selective reporting of results (domain 7) ask whether particular outcomes are reported from multiple outcome measures, particular analyses are reported from multiple analyses, and whether data are reported for only a subset of participants. We were unable to answer these questions unless the protocol for the study was available and published protocols are rare for observational studies. Therefore, we most frequently coded this domain as ‘not enough information’.	

### Practical considerations

Application of the ROBIN-E tool was time-consuming. First, time is required to prepare for coding by developing the tables of critical confounders and agreeing on criteria for valid measurements. The amount of time varies depending on the complexity of studies, but, as noted above, it took 2 weeks to develop the table of critical confounders for one systematic review. Regardless of the level of experience of the reviewer, it took 1 to 2.5 hours to code each paper. The tool was cumbersome to use because (1) the skip patterns were difficult to follow, (2) double negatives made answering yes or no confusing, (3) the language was often dense or overly complicated and (4) there were inconsistencies between words used in the tool and manual.

Most of the studies we rated with ROBINS-E were cohort studies, which is the observational design most similar to the RCT. We found that the applicability of the tool was worse for case-control studies. For example, the tool provides very limited guidance concerning key biases in the selection of cases and controls. Raters are asked only whether the population that gave rise to the cases was clearly defined, and not about other biases that might affect case selection, including whether there was full ascertainment, questionable exclusions or inclusion of irrelevant cases.

We do not discuss differences in terminology which the ROBINS-E developers acknowledge and which have been discussed extensively [1].

## Discussion

The ROBINS-E tool has been developed by consensus of an international team of investigators and has a number of strengths including providing a structured and transparent method to assess risk of bias in observational studies. We applied the tool to over 70 observational studies and found serious limitations. The premise that observational studies should be compared to the ‘ideal’ randomised controlled trial does not adequately capture all the sources of bias that should be considered for observational studies. Important questions related to assessing bias due to confounding and exposure assessment are missing. The ROBINS-E tool uses a rating scheme to calculate an overall risk of bias which fails to discriminate between studies with a single risk of bias or multiple risks of bias. For example, a systematic review of venous thromboembolic (VTE) risks of drospirenone-containing oral contraceptives considered all population-based studies using administrative data to be at serious or critical risk of bias because some potential confounders, such as family history, were not recorded in administrative data [39]. These studies, which used methods considered as state-of-the-art in pharmacoepidemiology, were judged to be at similar overall risk of bias to a study on a selected cohort of women that relied on initial self-report of VTE, with potential exposure recall bias and failure to exclude VTE for reasons unrelated to contraceptive use, such as surgery, cancer or pregnancy [40]. Bias related to ‘co-exposures’ should be addressed under confounding and questions about ‘unintended exposures’ do not make sense. Since ROBINS-E is derived from a tool for assessing studies of interventions (ROBINS-I), we noted a number of instances where the wording of the signalling questions used to guide judgements in each domain could not apply to exposure studies. The application of ROBINS-E was time-consuming and confusing as the raters could not always agree on the meaning of the questions. Similarly, as noted during the development of ROBINS-E, based on the narrative responses to the signalling questions, the raters reported misunderstanding the concepts in the questions and the information in the studies [19]. Although the tool is still in development and users should access the latest version [18], it is critical that concerns are addressed early in the refinement process.

Our experience in applying the ROBINS-E tool raises concerns that have also been observed by those applying the Cochrane risk of bias tool for observational studies of interventions (ROBINS-I, formerly ACROBAT-NRSI) [20, 21]. These studies have noted that the signalling questions need clarification, the application is time-consuming and the tool lacks testing for different study designs and topic areas. A recent study comparing the ROBINS-I to two other tools for assessing risk of bias in observational studies found that users of the tool rated the ROBINS-I lowest for clarity of instructions, clarity of items and discriminating between high and low risk of bias studies and that the ROBINS-I required the most time for training and application [41]. The strength of our study is that we report on experience applying ROBINS-E to over 70 studies of two designs (cohort and case control) over 3 topic areas.

Based on our experience, we do not recommend ROBINS-E for evaluating risk of bias in observational studies of exposures. We are concerned that the risk of bias assessments may not be useful or believable to those working with observational data, including systematic reviewers and guideline developers. The ROBINS-E has been derived from the ROBINS-I and has not been developed with input from potential users of the tool in mind. The reliability and reproducibility of the assessments is likely to be compromised because of the lack of clarity of specific components and a lengthy and complex set of instructions for use. It is also unclear whether ROBINS-E would stand up to an empirical assessment of the association between included risk of bias criteria and effects on study outcomes. This means that studies with methodological characteristics rated as high risk of bias will overestimate or underestimate effects compared to studies with lower risks of bias (e.g. a lack of randomisation will overestimate drug efficacy).

Exposure studies are frequently used to estimate the chance of harm occurring, for example, adverse health effects related to chemical or drug exposures. By predictably rating observational studies that inform decision making as low quality (as compared to an ideal RCT) application of ROBINS-E could question the validity of estimates of the nature and extent of potential harm. Application of ROBINS-E could bolster arguments of industries claiming that the evidence base is too weak to support regulation or policies to reduce harmful exposures and will potentially undermine policies that can protect people from harm. Often, these products are already being used in the marketplace and exposures are ongoing in the population, so delaying action will threaten public health protection.

Assessing risk of bias in observational studies of exposures is a complex topic, and it may be difficult for any tool to incorporate some aspects that are essential to evaluating observational studies. Furthermore, a single tool used to address bias in different observational study designs, such as proposed by the ROBINS-E, may be unrealistic. Further study and collaboration will be required to develop a simpler, alternative tool that meets the needs of the environmental and public health community. We are not suggesting that the constraints of observational studies should lead to a lower standard in how risk of bias is assessed in observational studies compared to RCTs. We are proposing that risk of bias assessments for observational studies need to be meaningfully and rigorously aligned with the sources of bias in studies of ‘real-world’ exposures. Selection of the items for a risk of bias tool should be informed by empirical evidence of bias and conceptual considerations. For example, randomisation and blinding are part of the Cochrane risk of bias tool for randomised trials because there is evidence that inadequate application of these methods overestimates efficacy estimates [42, 43]. We recommend similar empirical tests of the association between methods and results for each risk of bias domain to be included in a tool for assessing observational studies. Thus, rather than developing a tool by modifying one for evaluating trials of interventions, development should start with systematic reviews of methodological studies assessing the association of study design characteristics with effect estimates.

An empirically based tool will be useful to systematic reviewers and public health guideline developers if it is simple to apply and developed with input from potential users. We recommend that development of an empirically based tool should involve getting feedback from a variety of stakeholders to define each item that will be included. For example, development of an empirically based tool for assessing bias in studies of harmful environmental and drug exposures should involve researchers in environmental epidemiology and pharmacoepidemiology to ensure that the language and definitions used are consistent with these fields of research. We recommend that the questions avoid over or double counting bias domains. Lastly, we recommend that the tool and guidance for use are available for free open access to facilitate use.

## Conclusions

Although the ROBINS-E tool has been developed based on tools that are commonly used for assessing risk of bias in studies included in clinical systematic reviews and guidelines, our experience suggests that it does not meet the need for an international standard for evaluating human observational studies for questions of harm relevant to public and environmental health. We propose starting with an assessment of the empirical basis for items that should be included in a tool for assessing risk of bias in observational studies. This evidence could then be presented to a wide variety of stakeholders to gather further feedback on refining items for the tool. A simpler, empirically based tool is more likely to be adopted by systematic reviewers, guideline developers, journal editors and researchers conducting observational studies of exposures.

## References

[1] National Research Council (U.S.) (2014). Board on Environmental Studies and Toxicology. Committee to Review the IRIS Process: Review of EPA’s integrated risk information system (IRIS) process.

[2] Rooney AA, Boyles AL, Wolfe MS, Bucher JR, Thayer KA (2014). Systematic review and evidence integration for literature-based environmental health science assessments. Environ Health Perspect.

[3] United States. Congress. House. Committee on energy and commerce. Subcommittee on environment and the economy (2015). H.R. ______, the TSCA modernization act of 2015: hearing before the subcommittee on environment and the economy of the committee on energy and commerce, house of representatives, one hundred fourteenth congress, first session, April 14, 2015.

[4] 2016 NHMRC Standards for Guidelines [https://www.nhmrc.gov.au/guidelinesforguidelines/standards].

[5] Johnson PI, Koustas E, Vesterinen HM, Sutton P, Atchley DS, Kim AN, Campbell M, Donald JM, Sen S, Bero L (2016). Application of the navigation guide systematic review methodology to the evidence for developmental and reproductive toxicity of triclosan. Environ Int.

[6] Lawrence M, Naude C, Armstrong R, Bero L, Covic N, Durao S, Ghersi D, Macdonald G, MacLehose H, Margetts B (2016). A call to action to reshape evidence synthesis and use for nutrition policy. Cochrane Database Syst Rev.

[7] Morgan RL, Thayer KA, Bero L, Bruce N, Falck-Ytter Y, Ghersi D, Guyatt G, Hooijmans C, Langendam M, Mandrioli D (2016). GRADE: assessing the quality of evidence in environmental and occupational health. Environ Int.

[8] Vandenberg LN, Agerstrand M, Beronius A, Beausoleil C, Bergman A, Bero LA, Bornehag CG, Boyer CS, Cooper GS, Cotgreave I (2016). A proposed framework for the systematic review and integrated assessment (SYRINA) of endocrine disrupting chemicals. Environ Health.

[9] Woodruff TJ, Sutton P (2014). The navigation guide systematic review methodology: a rigorous and transparent method for translating environmental health science into better health outcomes. Environ Health Perspect.

[10] Higgins J, Green S (2008). Cochrane Handbook for Systematic Reviews of Interventions.

[11] Meerpohl JJ, Naude CE, Garner P, Mustafa RA, Schunemann HJ (2017). Comment on “perspective: NutriGrade: a scoring system to assess and judge the meta-evidence of randomized controlled trials and cohort studies in nutrition research”. Adv Nutr.

[12] Rooney AA, Cooper GS, Jahnke GD, Lam J, Morgan RL, Boyles AL, Ratcliffe JM, Kraft AD, Schunemann HJ, Schwingl P (2016). How credible are the study results? Evaluating and applying internal validity tools to literature-based assessments of environmental health hazards. Environ Int.

[13] Qaseem A, Forland F, Macbeth F, Ollenschlager G, Phillips S, van der Wees P (2012). Board of Trustees of the Guidelines International N: Guidelines International Network: toward international standards for clinical practice guidelines. Ann Intern Med.

[14] Higgins JP, Altman DG, Gotzsche PC, Juni P, Moher D, Oxman AD, Savovic J, Schulz KF, Weeks L, Sterne JA (2011). The Cochrane Collaboration’s tool for assessing risk of bias in randomised trials. BMJ.

[15] Savovic J, Weeks L, Sterne JA, Turner L, Altman DG, Moher D, Higgins JP (2014). Evaluation of the Cochrane Collaboration’s tool for assessing the risk of bias in randomized trials: focus groups, online survey, proposed recommendations and their implementation. Systematic reviews.

[16] Whiting PF, Rutjes AW, Westwood ME, Mallett S, Deeks JJ, Reitsma JB, Leeflang MM, Sterne JA, Bossuyt PM, Group Q (2011). QUADAS-2: a revised tool for the quality assessment of diagnostic accuracy studies. Ann Intern Med.

[17] Sterne JA, Hernan MA, Reeves BC, Savovic J, Berkman ND, Viswanathan M, Henry D, Altman DG, Ansari MT, Boutron I (2016). ROBINS-I: a tool for assessing risk of bias in non-randomised studies of interventions. BMJ.

[18] Preliminary risk of bias for exposures tool template [http://www.bristol.ac.uk/population-health-sciences/centres/cresyda/barr/riskofbias/robins-e/].

[19] Morgan RL, Thayer KA, Santesso N, Holloway AC, Blain R, Eftim SE, Goldstone AE, Ross P, Guyatt G, Schunemann HJ (2018). Evaluation of the risk of bias in non-randomized studies of interventions (ROBINS-I) and the ‘target experiment’ concept in studies of exposures: rationale and preliminary instrument development. Environ Int.

[20] Bilandzic A, Fitzpatrick T, Rosella L, Henry D (2016). Risk of bias in systematic reviews of non-randomized studies of adverse cardiovascular effects of thiazolidinediones and cyclooxygenase-2 inhibitors: application of a new Cochrane risk of bias tool. PLoS Med.

[21] Thomson H, Craig P, Hilton-Boon M, Campbell M, Katikireddi SV (2018). Applying the ROBINS-I tool to natural experiments: an example from public health. Systematic reviews.

[22] Praagman J, Franco OH, Ikram MA, Soedamah-Muthu SS, Engberink MF, van Rooij FJ, Hofman A, Geleijnse JM (2015). Dairy products and the risk of stroke and coronary heart disease: the Rotterdam study. Eur J Nutr.

[23] Johannes CB, Varas-Lorenzo C, McQuay LJ, Midkiff KD, Fife D (2010). Risk of serious ventricular arrhythmia and sudden cardiac death in a cohort of users of domperidone: a nested case-control study. Pharmacoepidemiol Drug Saf.

[24] Nissenbaum MA, Aramini JJ, Hanning CD (2012). Effects of industrial wind turbine noise on sleep and health. Noise Health.

[25] Harris PA, Taylor R, Thielke R, Payne J, Gonzalez N, Conde JG (2009). Research electronic data capture (REDCap)--a metadata-driven methodology and workflow process for providing translational research informatics support. J Biomed Inform.

[26] Hernan MA, Alonso A, Logan R, Grodstein F, Michels KB, Willett WC, Manson JE, Robins JM (2008). Observational studies analyzed like randomized experiments: an application to postmenopausal hormone therapy and coronary heart disease. Epidemiology.

[27] Io M (2008). Knowing what works in health care: a roadmap for the nation.

[28] Anglemyer A, Horvath HT, Bero L (2014). Healthcare outcomes assessed with observational study designs compared with those assessed in randomized trials. Cochrane Database Syst Rev.

[29] Golder S, Loke YK, Bland M (2011). Meta-analyses of adverse effects data derived from randomised controlled trials as compared to observational studies: methodological overview. PLoS Med.

[30] Elwood M (1998). Critical appraisal of epidemiological studies and clinical trials.

[31] Blair A, Stewart P, Lubin JH, Forastiere F (2007). Methodological issues regarding confounding and exposure misclassification in epidemiological studies of occupational exposures. Am J Ind Med.

[32] Endometrial cancer and combined oral contraceptives (1988). The who collaborative study of neoplasia and steroid contraceptives. Int J Epidemiol.

[33] Whiting P, Savovic J, Higgins JP, Caldwell DM, Reeves BC, Shea B, Davies P, Kleijnen J, Churchill R, Group R (2016). ROBIS: a new tool to assess risk of bias in systematic reviews was developed. J Clin Epidemiol.

[34] Lam J, Sutton P, Kalkbrenner A, Windham G, Halladay A, Koustas E, Lawler C, Davidson L, Daniels N, Newschaffer C (2016). A systematic review and meta-analysis of multiple airborne pollutants and autism spectrum disorder. PLoS One.

[35] Juni P, Witschi A, Bloch R, Egger M (1999). The hazards of scoring the quality of clinical trials for meta-analysis. Jama.

[36] Herbison P, Hay-Smith J, Gillespie WJ (2006). Adjustment of meta-analyses on the basis of quality scores should be abandoned. J Clin Epidemiol.

[37] Lundh A, Lexchin J, Mintzes B, Schroll JB, Bero L (2017). Industry sponsorship and research outcome. Cochrane Database Syst Rev.

[38] White J, Bero LA (2010). Corporate manipulation of research: strategies are similar across five industries. Stanford Law Policy Rev.

[39] Larivee N, Suissa S, Khosrow-Khavar F, Tagalakis V, Filion KB (2017). Drospirenone-containing oral contraceptive pills and the risk of venous thromboembolism: a systematic review of observational studies. BJOG.

[40] Dinger JC, Heinemann LA, Kuhl-Habich D (2007). The safety of a drospirenone-containing oral contraceptive: final results from the European Active Surveillance Study on oral contraceptives based on 142,475 women-years of observation. Contraception.

[41] Losilla JM, Oliveras I, Marin-Garcia JA, Vives J (2018). Three risk of bias tools lead to opposite conclusions in observational research synthesis. J Clin Epidemiol.

[42] Wood L, Egger M, Gluud LL, Schulz KF, Juni P, Altman DG, Gluud C, Martin RM, Wood AJ, Sterne JA (2008). Empirical evidence of bias in treatment effect estimates in controlled trials with different interventions and outcomes: meta-epidemiological study. BMJ.

[43] Page MJ, Higgins JP, Clayton G, Sterne JA, Hrobjartsson A, Savovic J (2016). Empirical evidence of study design biases in randomized trials: systematic review of meta-epidemiological studies. PLoS One.

